# Pneumocystis Pneumonia Presenting With Peripheral Predominant Consolidation and Traction Bronchiectasis

**DOI:** 10.7759/cureus.63257

**Published:** 2024-06-26

**Authors:** Nobuhiro Fujishima, Yoshihide Hioki, Atsushi Yokoyama, Kazufumi Hiramatsu, Kosaku Komiya

**Affiliations:** 1 Respiratory Medicine and Infectious Diseases, Oita University, Yufu, JPN

**Keywords:** organizing pneumonia, pneumocystis pneumonia, interstitial pneumonia, human immunodeficiency virus, acquired immunodeficiency syndrome

## Abstract

*Pneumocystis jirovecii* pneumonia (PCP) typically presents as a predominant ground-glass opacity (GGO) in the upper lobes. We report a case of a patient with PCP that mimicked organizing pneumonia or nonspecific interstitial pneumonia, showing peripheral predominant consolidation with traction bronchiectasis and peribronchovascular thickening in the lower lobes on high-resolution computed tomography (HRCT). *Pneumocystis jirovecii* was detected in bronchoalveolar lavage (BAL), and no other pathogens were isolated. After confirmation of a high plasma human immunodeficiency virus (HIV)-RNA titer and a low CD4+ cell count, the patient was diagnosed with PCP associated with HIV infection. The peripheral predominant consolidation was successfully resolved after treatment with trimethoprim-sulfamethoxazole. To the best of our knowledge, no previous case of PCP presenting with peripheral predominant consolidation, traction bronchiectasis, or peribronchovascular thickening has been reported. Physicians should consider PCP as a differential diagnosis even in cases suspected as organizing pneumonia or nonspecific interstitial pneumonia on HRCT.

## Introduction

Pneumocystis pneumonia (PCP) is a fatal pulmonary infection caused by the fungus *Pneumocystis jirovecii*. Most PCP cases develop in immunocompromised hosts, including those with human immunodeficiency virus (HIV) infection, malignancies, post-organ transplantation, and the use of immunosuppressive agents [1]. Patients with PCP usually present with symptoms such as fever, nonproductive cough, malaise, and progressive dyspnea [2]. High-resolution computed tomography (HRCT) typically reveals extensive ground-glass opacity (GGO), predominantly in the perihilar areas of the upper lobes of both lungs [2, 3]. If GGO or consolidation is distributed in peripheral areas, it may lead to a misdiagnosis, such as interstitial pneumonia, without considering PCP. Delayed diagnosis should be avoided to ensure prompt initiation of treatment for PCP and to screen for underlying immunosuppressive conditions, including HIV infection.

In this case report, we present a case of PCP with peripheral predominant consolidation accompanied by traction bronchiectasis and peribronchovascular thickening, which mimicked organizing pneumonia or nonspecific interstitial pneumonia pattern on HRCT.

## Case presentation

A man in his 60s without a significant medical history, including immunosuppressive medications, presented to a prior hospital due to fatigue and dyspnea that had persisted for three months. A chest X-ray revealed peripheral predominant consolidation in the lower lobes of the lungs, leading to a suspicion of interstitial pneumonia. For further evaluation, he was referred to our hospital within a few days.

On physical examination, his vital signs were as follows: body temperature of 37.2°C, oxygen saturation level (SpO_2_) of 93% on room air, respiratory rate of 18 breaths per minute, blood pressure of 86/60 mmHg, and heart rate of 105 beats per minute. Fine crackles were heard on auscultation in both lower lungs. Arterial blood gas analysis, performed without oxygen supplementation, indicated mild hypoxia (acid-base balance of the blood (pH): 7.470; partial pressure of oxygen (PaO_2_): 53 Torr; partial pressure of carbon dioxide (PaCO_2_): 31 Torr; bicarbonate (HCO3): 22.6 mmol/L). Laboratory blood tests revealed elevated levels of C-reactive protein (3.99 mg/dL), aspartate aminotransferase (71.8 IU/L), and lactate dehydrogenase (318 IU/L). The patient also had low levels of white blood cells (2,890 per μL) and hemoglobin (11.4 g/dL), while blood urea nitrogen (14.5 mg/dL) and serum creatinine (0.63 mg/dL) were within normal ranges. The KL-6 level was elevated (860 U/mL). Antibodies specific to collagen vascular diseases, including anti-cyclic citrullinated peptide antibodies, anti-Jo 1 antibodies, anti-ribonucleoprotein antibodies, anti-double stranded DNA antibodies, and anti-SS-A/Ro antibodies, were all negative, except for proteinase-3-antineutrophil cytoplasmic antibody (PR3-ANCA), which was slightly elevated (14.3 U/mL). However, his urinalysis resulted in normal results, and no other clinical features of suspected ANCA-associated vasculitis were observed.

**Figure 1:**
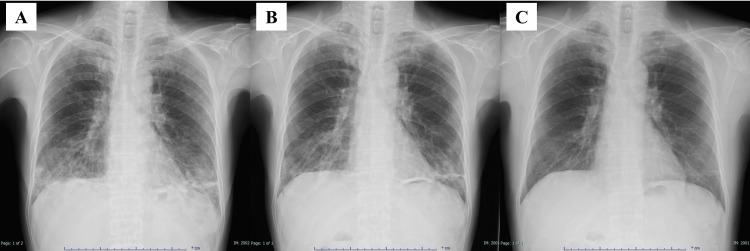
Chest X-ray on admission showing lung infiltration predominantly in the lower lung fields (A), and chest X-ray 62 days (B) and three months (C) after hospitalization showing further improvement.

**Figure 2:**
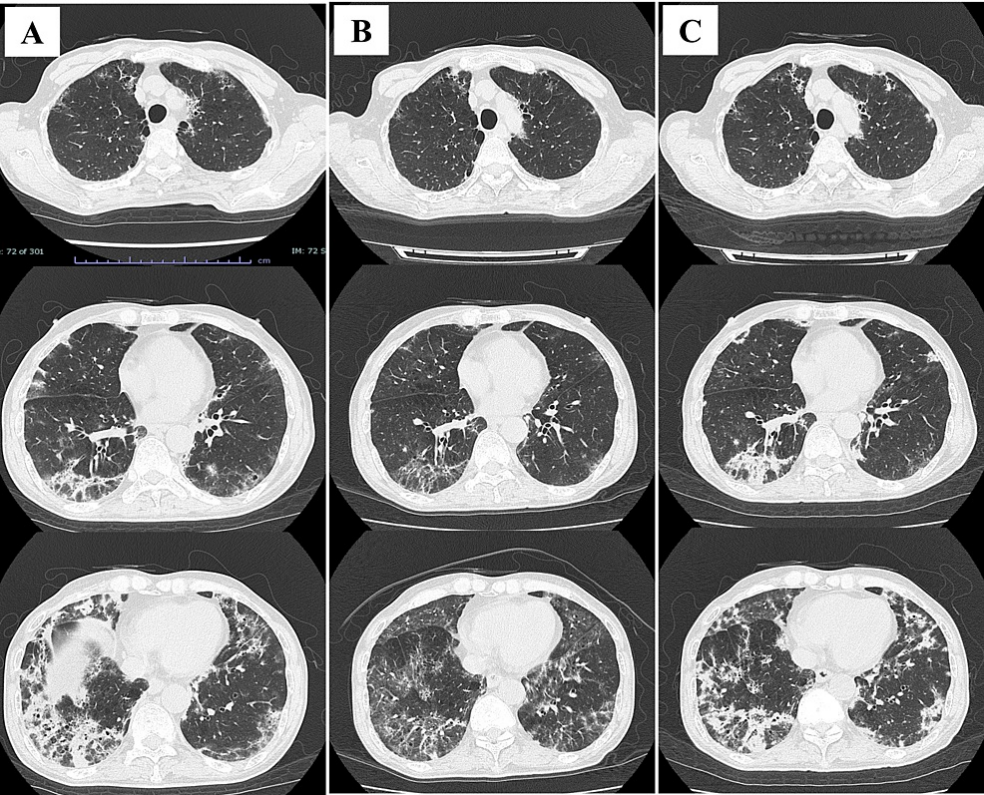
High-resolution computed tomography (HRCT), performed two days after hospitalization, revealed peripheral predominant consolidation with traction bronchiectasis and peribronchovascular thickening in the lower lobes (A). An HRCT performed 22 days after hospitalization indicated improvement in consolidation (B). An HRCT performed 35 days after hospitalization showed re-enhancement of the consolidation (C).

A chest X-ray taken upon admission to our hospital showed lung infiltration predominantly in the lower lung fields (Figure 1A). A chest HRCT revealed peripheral predominant consolidation with traction bronchiectasis and peribronchovascular thickening in the lower lobes of both lungs (Figure 2A). *Pneumocystis jirovecii* was detected by polymerase chain reaction in bronchoalveolar lavage (BAL), although the Grocott staining test was negative. No other pathogens were isolated in the culture. The level of beta-D-glucan was slightly elevated (22.3 pg/mL). A transbronchial lung biopsy could not be performed due to the patient’s poor oxygenation status. We diagnosed the patient with PCP and started treatment with trimethoprim-sulfamethoxazole (720/3600 mg/day) on the third day after admission. Oral prednisolone (60 mg/day) was concurrently administered as his PaO_2_ was <70 mmHg [4].

An HIV screening test performed six days after admission was positive, revealing a high HIV-RNA titer (3.1 × 105 copies/mL) and a low CD4+ cell count (4 cells/μL). Although the patient tested weakly positive for cytomegalovirus antigenemia, no findings suggestive of cytomegalovirus infection were observed in the BAL fluid. Therefore, treatment for cytomegalovirus was not initiated. Due to side effects of the medication, including hyponatremia, hyperkalemia, leukopenia, thrombocytopenia, and hepatic dysfunction, trimethoprim-sulfamethoxazole was terminated on day 21. At the moment, while the KL-6 level was further elevated (1243 U/mL), the beta-D glucan level was dropped to the normal range (less than 3.0 pg/mL). An HRCT performed the next day indicated improvements in the consolidation (Figure 2B).

On day 24, the patient was diagnosed with cytomegalovirus retinitis due to worsening cytomegalovirus antigenemia, and treatment with valganciclovir was immediately started. Treatment with tenofovir/emtricitabine and raltegravir potassium for HIV infection also started on the same day. However, a slight fever was noted, and a chest HRCT revealed a re-enhancement of the consolidation on day 35 (Figure 2C). While KL-6 levels did not significantly change (1018 U/mL), beta-D glucan levels were significantly elevated (98.4 pg/mL). Since BAL did not indicate evidence of any infection, the patient was considered to have an immune reconstitution inflammatory syndrome complicated with PCP. Treatment with pentamidine was started, but it was switched to atovaquone due to hypoglycemia on day 44. The lung infiltration improved (Figure 1B), and the patient was discharged on day 65 and continued on an outpatient basis. A chest X-ray taken three months after discharge from the hospital showed further improvement (Figure 1C).

## Discussion

We experienced a case of PCP associated with HIV infection, initially suspected as interstitial pneumonia due to the presence of peripheral predominant consolidation with traction bronchiectasis and peribronchovascular thickening. Typically, HRCT in patients with PCP reveals a central, predominant distribution of GGO in the upper lobes of both lungs. This pattern is believed to be due to the preference of the fungus for lung areas that are better ventilated, have higher oxygen tension, and have less effective lymphatic drainage [5]. There have been no prior reports of PCP presenting as peripheral predominant consolidation mimicking interstitial pneumonia.

The major determinant of lung injury patterns in PCP is considered to be the inflammatory response of the host rather than the virulence of the infecting organism itself [6]. A study reported that HRCT features in patients with PCP with HIV infection are associated with CD4+ cell count [7]. Higher CD4+ cell counts result in a predominance of GGOs, whereas lower CD4+ cell counts result in more prominent cysts. In this case, the CD4+ cell count was extremely low (4 cells/µL), which might have contributed to the atypical radiological features.

Moreover, the interval between symptom onset and hospital visit may affect the radiological features. For example, PCP often presents as GGO in the early phase, but consolidation can develop in the advanced phase [3, 8]. The median time from symptom onset to diagnosis of PCP in patients with HIV infection is 21 days [6]. In this case, the diagnosis was made four months after the onset of symptoms. Although no studies have specifically investigated the association between the interval from symptom onset to diagnosis and the distribution of lung infiltrations, the delayed diagnosis in this case might have influenced the distribution of lung field consolidation. There were case reports of organizing pneumonia associated with *Pneumocystis jiroveci* immune reconstitution inflammatory syndrome in HIV-positive patients [9]. The peripheral dominant consolidation in the current case appeared to be an organizing pneumonia pattern on HRCT. However, it was observed before the treatment for HIV infection started. The complexity of immune responses in patients with HIV infection may have the potential to affect these abnormal radiological manifestations.

In addition, it is important to consider whether the patient was also complicated with interstitial pneumonia, such as organizing pneumonia or nonspecific interstitial pneumonia. In such cases, consolidation on HRCT is often distributed predominantly peripherally [10], and these conditions are frequently associated with collagen vascular diseases [11]. Indeed, HIV infection is known to be more frequently complicated by autoimmune diseases [12]. For example, it is estimated that 13%-83% of HIV patients are ANCA-positive [13]. The patient in the current case had a positive result for PR3-ANCA without any features of ANCA-associated vasculitis. However, the peripheral consolidation may have been linked to ANCA-associated interstitial pneumonia. This seems less likely because the PR3-ANCA turned negative after hospital discharge.

The lesson learned from this case report is that PCP should be suspected even when HRCT presents peripheral predominant consolidation, as such cases may be misdiagnosed as interstitial pneumonia. While the radiological presentation of PCP is diverse, no previous cases have reported consolidation predominantly in the peripheral lower lobes of both lungs. A limitation of this case is that a lung biopsy was not performed, leaving it unclear whether the patient had features of *Pneumocystis jirovecii* or ANCA-associated vasculitis in the tissue. Moreover, since the patient was treated with prednisolone for PCP due to low oxygenation status, it was difficult to conclude that PCP was the sole phenomenon of the disease. Prednisolone of 80 mg per day is usually recommended for the initial dose when patients with PCP and low oxygenation status are initially treated [4], but we administered the reduced dose (60 mg per day) because of his low body mass index (16.7 kg/m2).

## Conclusions

When HRCT shows peripherally distributed consolidation with traction bronchiectasis and peribronchovascular thickening, interstitial pneumonia is often diagnosed. However, the possibility of atypical cases of PCP needs to be considered. Further research focusing on the distribution of lung involvement is required to determine the factors associated with atypical radiological features in patients with PCP.
